# Functional tailoring of a PET hydrolytic enzyme expressed in *Pichia pastoris*

**DOI:** 10.1186/s40643-023-00648-1

**Published:** 2023-04-06

**Authors:** Xian Li, Beilei Shi, Jian-Wen Huang, Ziyin Zeng, Yu Yang, Lilan Zhang, Jian Min, Chun-Chi Chen, Rey-Ting Guo

**Affiliations:** State Key Laboratory of Biocatalysis and Enzyme Engineering, Hubei Hongshan Laboratory, Hubei Collaborative Innovation Center for Green Transformation of Bio-Resources, Hubei Key Laboratory of Industrial Biotechnology, School of Life Sciences, Hubei University, Wuhan, 430062 People’s Republic of China

**Keywords:** Polyethylene terephthalate, Biodegradation, PET hydrolase, *Caldimonas taiwanensis*, *Pichia pastoris*, *N-*glycosylation, Enzyme engineering

## Abstract

Using enzymes to hydrolyze and recycle poly(ethylene terephthalate) (PET) is an attractive eco-friendly approach to manage the ever-increasing PET wastes, while one major challenge to realize the commercial application of enzyme-based PET degradation is to establish large-scale production methods to produce PET hydrolytic enzyme. To achieve this goal, we exploited the industrial strain *Pichia pastoris* to express a PET hydrolytic enzyme from *Caldimonas taiwanensis* termed *Ct*PL-DM. In contrast to the protein expressed in *Escherichia coli*, *Ct*PL-DM expressed in *P. pastoris* is inactive in PET degradation. Structural analysis indicates that a putative *N*-glycosylation site N181 could restrain the conformational change of a substrate-binding Trp and hamper the enzyme action. We thus constructed N181A to remove the *N*-glycosylation and found that the PET hydrolytic activity of this variant was restored. The performance of N181A was further enhanced via molecular engineering. These results are of valuable in terms of PET hydrolytic enzyme production in industrial strains in the future.

**Graphical Abstract:**

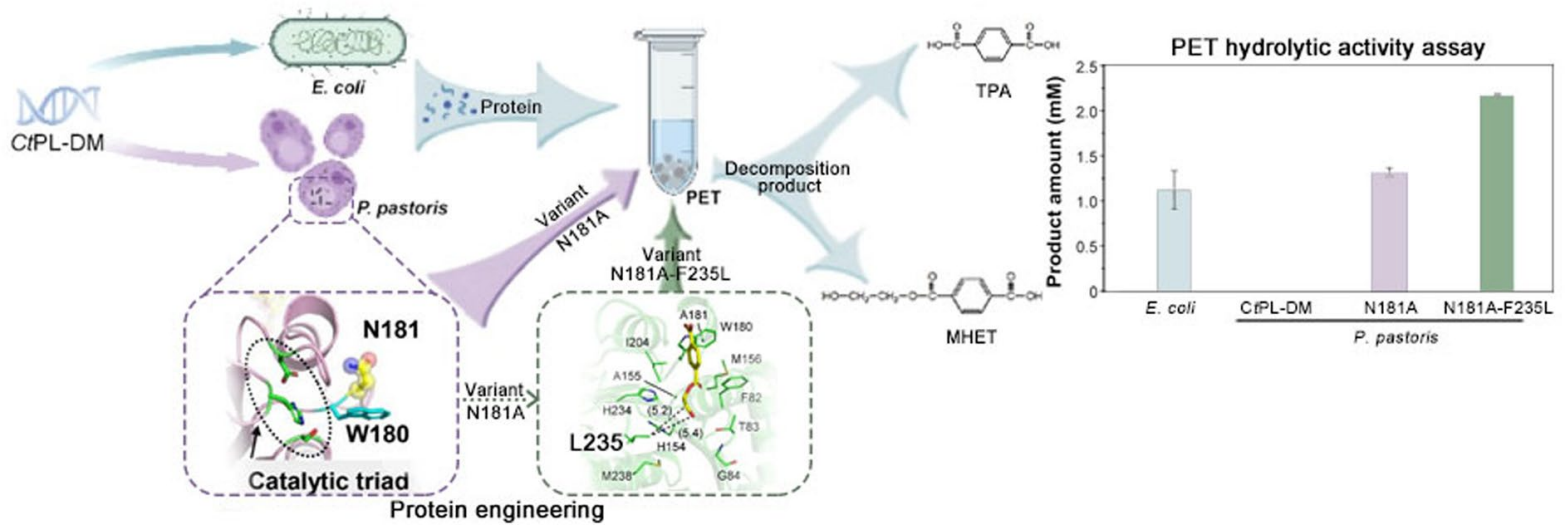

**Supplementary Information:**

The online version contains supplementary material available at 10.1186/s40643-023-00648-1.

## Introduction

The accumulation of polyethylene terephthalate (PET) wastes has become a severe burden to the global environment and ecosystem (Lear et al. 2021). Enzyme-based degradation of PET is an eco-friendly recycling strategy compared with other waste management methods such as chemical and mechanical processes. In recent years, many microbial strains and enzymes that can degrade PET have been discovered. However, these machineries often exhibit poor activity, such that numerous engineering efforts have been paid to improve their efficacy (Urbanek et al. 2021; Zhu et al. 2022; Kawai 2021). Furthermore, PET monomeric constituent terephthalic acid (TPA) yielded by the engineered PET degrading enzymes can be recovered and used for the re-synthesis of PET, suggesting that the bio-based PET recycling process can be practiced in principle (Tournier et al. 2020; Lu et al. 2022; Dissanayake and Jayakody 2021).

A number of enzymes that exhibit PET degradation activity have been reported, which mainly are ester bond hydrolytic enzymes including cutinases, lipases, and esterases (Kawai et al. 2019; Gao et al. 2021). In 2016, Yoshida et al. isolated a PET assimilating bacterium *Ideonella sakaiensis* 201-F6 from a PET recycling site, which exploits a dual-enzyme system (*Is*PETase and *Is*MHETase) to breakdown PET into TPA and ethylene glycol (EG) (Yoshida et al. 2016). *Is*PETase is the first naturally evolved PET hydrolytic enzyme, which shares a high sequence identity to cutinases and shows superior PET degradation activity at a moderate temperature in comparison with other PET degrading enzymes. The crystal structure of *Is*PETase was soon after reported by several groups, which revealed unique features of the enzyme in having a flexible substrate-binding pocket conferred by the wobbling TPA-interacting residue W185 (Austin et al. 2018; Chen et al. 2018; Fecker et al. 2018; Joo et al. 2018; Liu et al. 2019a). Recently, we found that the W185 wobbling is resulted from the presence of two *Is*PETase-unique amino acids (S214 and I218) that are located under W185, whose equivalents are His and Phe in all homologous enzymes (Chen et al. 2021). Substituting His/Phe with Ser/Ile should expand the substrate-binding pocket to accommodate the bulkier substrate PET, and the strategy termed “double mutation (DM)” has proven to be useful to enhance PET hydrolytic activity of several *Is*PETase-like enzymes.

High-level production and good performance characteristics of PET degrading enzymes are required to facilitate the industrial applications of these enzymes. Several expression systems have been reported for heterologous expression of PET degrading enzymes. *Escherichia coli* intracellular expression is one of the most commonly used technique in laboratory due to low cost and ease of manipulation, but further applications with this system are hampered by additional protein purification processes. Secreted expression in *E. coli* involving signal peptide optimization and chaperon co-expression was thus exploited for *Is*PETase production, but the bacterial endotoxin remains a potential threat (Seo et al. 2019; Shi et al. 2021; Aer et al. 2022). Other prokaryotic cell factories such as *Bacillus subtilis* and *Pseudomonas putida* have been utilized to scale up PET hydrolase production (Xi et al. 2021; Wang et al. 2020; Oh et al. 2022; Huang et al. 2018). Microalgae are also attractive hosts because of its rich resources and wide utilizations in many areas and commercial applications. A diatom *Phaeodactylum tricornutum* and green algae, *Chlamydomonas reinhardtii* were also employed to functionally express *Is*PETase, suggesting that it may be a potential industrial application strategy of PET degradation (Kim et al. 2020).

*Pichia pastoris* is among the most widely applied industrial strains for enzyme production, which is advantageous in easy gene manipulation, secretory expression, low production cost, and fast growth speed. Complex post-translational modifications can be achieved in *P. pastoris*, therefore large quantities of recombinant protein with correct folding, disulfide bond formation as well as glycosylation, can be obtained (Yang and Zhang 2018). Moreover, large amounts of cells can be easily grown in minimal medium through fed-batch high-cell-density fermentation methodology (Liu et al. 2019b). With this expression system, a highly pure demanded protein can be produced without further purification procedures owing to the low basal level endogenous secretory proteins (Karbalaei et al. 2020). We have also demonstrated that *P. pastoris* can be used to express *Is*PETase and a *Is*PETase-like enzyme termed *Bur*PL, which exhibit better thermostability than those expressed in *E. coli* (Chen et al. 2021; Shirke et al. 2018).

Notable, the expression and function of prokaryotic proteins might be compromised to various extents when expressed in the eukaryotic system. This can be overcome by optimization processes, including codon optimization, promoter strengthening, chaperone co-expression, and protein engineering (Juturu and Wu 2018; Jariyachawalid et al. 2012). As shown in our previous study, the *N-*linked glycosylation of the *P. pastoris*-expressed *Is*PETase and *Bur*PL appear to pose negative effects on their PET hydrolytic activity and de-glycosylation treatment can significantly elevate the enzyme activity (Chen et al. 2021). This phenomenon has also been reported elsewhere that *N*-glycosylations in eukaryotic expression systems could interfere the bio-activity of prokaryotic genes, and de-glycosylation or mutagenesis can be applied to restore the protein function (Tarahomjoo et al. 2008; Huang et al. 2013; Han et al. 2020).

Here, we report our experience in establishing the *P. pastoris* strain that expresses a PET hydrolytic enzyme from *Caldimonas taiwanensis* termed *Ct*PL-DM that was identified from sequence mining from GenBank and modified by the DM strategy. This enzyme exhibits optimal activity at 60 ℃ and shows application potentials in PET biodegradation. The process to enhance protein expression and activity of *Ct*PL-DM in *P. pastoris* will be demonstrated, which should be of great value to guide further industrial applications of PET hydrolytic enzymes.

## Material and methods

### Plasmid construction and mutagenesis

Plasmid that carries the coding gene of *Ct*PL-DM has been described previously (Chen et al. 2021). The target gene was cloned to pET32a and pPICZαA vectors for enzyme expression in *E. coli* and *P. pastoris,* respectively.

The variants were generated through PCR-based site-directed mutagenesis with the pPICZαA carrying wild-type *Ct*PL-DM as a template. The sequences of the mutagenesis oligonucleotides are listed in Additional file 1: Table S1. The PCR reaction consisting of 2 × Phanta Flash Master Mix (12.5 μL), 0.5 μL forward primer, 0.5 μL reverse primer, 0.5 μL template DNA, 11 μL double distilled water was subjected to amplification with the following program: 95 °C for 3 min, 25 cycles (95 °C for 30 s, 55 °C for 30 s, 72 °C for 1 min), followed by the final extension at 72 °C for 5 min. The PCR products were digested with *Dpn*I to remove the template DNA, and then transformed into *E. coli* DH5α cells with selection for resistance to ampicillin (100 mg L^−1^). All constructed plasmids were validated by direct DNA sequencing, and the mutants were expressed in *P. pastoris* and purified as *Ct*PL-DM, the crystallization and enzyme activity measurement were conducted according to the methods described in the following paragraphs.

### Recombinant protein expression and purification in *E. coli*

The pET32a plasmid carrying *Ct*PL-DM was transformed into *E. coli* BL21 (DE3) and grown in Luria–Bertani medium containing 100 mg L^−1^ ampicillin at 37 °C. When OD600 reached ~ 0.8, the protein expression was induced with 0.3 mM IPTG at 16 °C for 18 h. The cells were harvested by centrifugation at 6000 × *g* for 10 min and then resuspended in buffer A that contains 25 mM Tris–HCl, 150 mM NaCl and 20 mM imidazole (pH 7.5). Cells were then disrupted with a French press, and cell debris was removed by centrifugation at 27,000 × *g* and 4 °C for 1 h. The supernatant was then applied to a buffer A-equilibrated Ni–NTA column and eluted against a linear gradient of 0 to 60% buffer B (25 mM Tris–HCl, 150 mM NaCl and 500 mM imidazole, pH 7.5) at a flow rate of 4 mL min^−1^. The target protein-containing fractions were collected and dialyzed against buffer C (25 mM Tris–HCl, 150 mM NaCl, pH 7.5) which contains tobacco etch virus (TEV) protease to remove the thioredoxin and His-tag. The protein-containing solution was then passed through a second Ni–NTA column and the untagged target protein in flow-through was collected, concentrated and stored at -80 °C before further analyses.

### Recombinant protein expression and purification in *P. pastoris*

The protein expression and purification processes of *Ct*PL-DM and variants follow the same procedures. The *Pme*I-linearized recombinant pPICZαA plasmids were transformed into *P. pastoris* X33 by electroporation. Transformants that carry the target gene were selected on YPD plates (1% yeast extract, 2% peptone, 2% dextrose, 2% agar) containing 250 μg mL^−1^ zeocin. Single colonies were inoculated in 5 mL YPD and cultured at 30 °C for 24 h prior to induction by BMMY (1% yeast extract, 2% peptone, 100 mM potassium phosphate (pH 6.0), 1.34% yeast nitrogen base with ammonium, 4 × 10^–5^% biotin and 0.5% methanol) and at 30 °C for 48 h. The protein expression in culture supernatant was examined by SDS-PAGE and the highest clones were chosen for further experiments. The cells were inoculated into 10 mL of YPD at 30 °C for 24 h and transferred to 500 mL BMGY (1% yeast extract, 2% peptone, 100 mM potassium phosphate (pH 6.0), 1.34% yeast nitrogen base with ammonium, 4 × 10^–5^% biotin and 1% glycerol) for another 24 h. Then the culture medium was replaced with 500 mL BMMY medium and 1% methanol was supplemented every 24 h to induce protein expression for five days. The supernatant was collected and concentrated by ~ tenfold using an ultrafiltration unit with a 10-kDa cutoff membrane (Sartorius Stedim Biotech) and then dialyzed against a buffer containing 25 mM HEPES (pH 7.0) at 4 °C for 24 h. Owing to lack of His-tag, supernatants were purified by employing ion exchange chromatography. Target protein-containing solution was applied onto an SP Sepharose column (GE Healthcare) and eluted with a NaCl gradient of 0–1 M at a flow rate of 4 mL min^−1^. The protein-containing fractions were collected, concentrated and stored at -80 °C before further analyses.

### Crystallization and structure determination

Crystallization trials were conducted with commercial crystallization kit via the method of sitting drop vapor-diffusion at 25 °C. The ratio of protein solution and reservoir solution is 1:1. The mixture in 96-well Cryschem plates and equilibrated against 100 μL reservoir solution. The optimal crystallization condition of each protein was as follows: *Ct*PL-DM, 0.1 M potassium bromide, 33% w/v PEG 2000 MME; *Ct*PL-DM-S155A, 20% PEG 2000 MME, 0.2 M ammonium citrate tribasic (pH 7.0), 0.1 M imidazole; *Ct*PL-DM-N181A-F235L-S155A, 0.8 M lithium chloride, 5% PEG 6000, 0.1 M citric acid (pH 4.0). The X-ray diffraction datasets were collected using a Bruker D8 Venture coupled with a CMOS-PHOTON II detector at Hubei University and processed by Proteum 3 (Bruker AXS GmbH). The structures of *Ct*PL-DM was first solved by molecular replacement method using the program PHASER in the CCP4i suite (Potterton et al. 2003) with a template built from *Is*PETase as a searching model. Subsequent model refinement was conducted with Coot (Emsley and Cowtan 2004) and Refmac5 (Murshudov et al. 1997). Prior to structure refinement, 5% randomly selected reflections were set aside for calculating *R*_free_ as a monitor of model quality. The refined structure of *Ct*PL-DM was used as a template to solve the other *Ct*PL-DM structures.

### PET degrading activity measurement

The PET hydrolytic activity of *Ct*PL-DM and variants was measured by the same procedures that was described previously with some modifications (Chen et al. 2021). 1-mL reaction mixtures containing 3 mg ground PET powder (Sigma-Aldrich, cat. no. 429252) or a piece of Goodfellow amorphous PET film (GfPET, 6 mm in diameter, ~ 8 mg, crystallinity of 7.3%), 10 μg mL^−1^ purified enzyme and 50 mM glycine–NaOH buffer (pH 9.0) were incubated at indicated temperatures with agitation at 800 rpm for various period of times. The reaction was terminated by heating at 85 °C for 10 min. The mixtures were then passed through a 0.22-μm filter and then analyzed by HPLC analytic system coupled with a C18 column. The mobile phase consists of 20 mM phosphate buffer (pH 2.5) and a methanol gradient from 35 to 70% in 0–25 min at a flow rate of 1 mL min^−1^ was used. The hydrolytic products MHET and TPA were recorded by monitoring the absorbance at 240 nm and their amounts were calculated based on standard curves generated from a series of MHET and TPA with known concentrations.

## Results and discussion

### Secretion expression of *Ct*PL-DM in *P. pastoris*

**Fig. 1:**
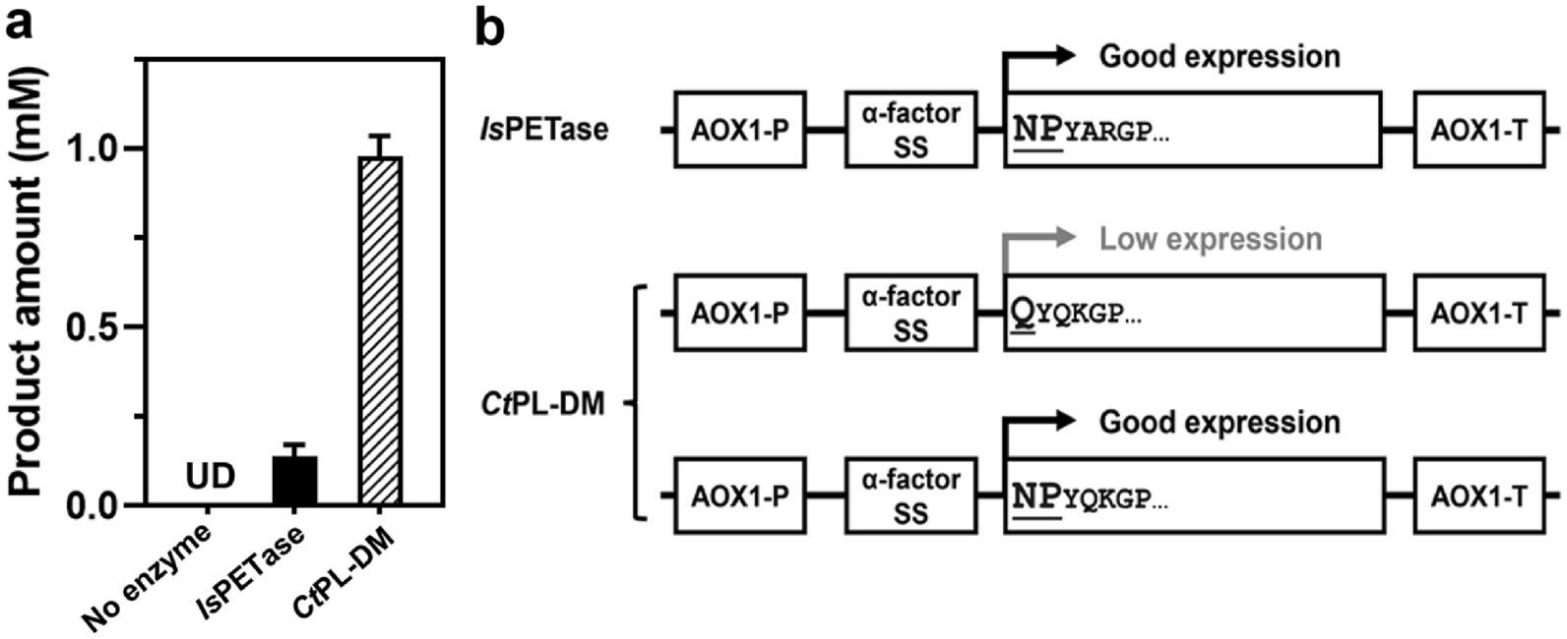
Comparison of *Is*PETase and *Ct*PL-DM.** a** Total released PET hydrolytic products of recombinant protein of *Is*PETase and *Ct*PL-DM expressed in *E. coli* by using GfPET as a substrate. The reaction temperatures of *Is*PETase and *Ct*PL-DM are 30 °C and 60 °C, respectively. Blank control that contained no enzyme in the reaction was also analyzed. A triplicate assay was conducted and the average values ± standard deviation are presented. **b** Schematic representation of *P. pastoris* expression units. The structures of expression unit of pPICZαA vectors carrying *Is*PETase and *Ct*PL-DM are depicted. The amino acids in the N-terminal part of each expressing sequences were displayed. AOX1-P, *P. pastoris* alcohol oxidase 1 promoter; α-factor SS, α-factor secretion signal; AOX1-T, *P. pastoris* alcohol oxidase 1 terminator

Our previous report indicated that *Ct*PL-DM exhibits potent PET hydrolytic activity that about sevenfold more hydrolytic products were obtained from hydrolyzing GfPET in comparison with *Is*PETase (Fig. 1a). We then aimed to explore the industrial application potentials of *Ct*PL-DM by employing *P. pastoris* as the protein expression vehicle. Unfortunately, our initial attempt to express *Ct*PL-DM in *P. pastoris* was not successful that extremely low, if any, protein expression was detected (data not shown). This is in stark contrast to our experiences with *Is*PETase and *Bur*PL (Chen et al. 2021). Through inspecting the sequences of *Is*PETase and *Ct*PL-DM in the recombinant pPICZαA plasmids, we noticed that the amino acids in the conjugation region between the secretion signal and the mature sequence of two constructs are different (Fig. 1b). We suspected that the inefficient cleavage of the secretion signal might lead to low protein expression in the original *Ct*PL-DM construct and thus altered the N-terminal amino acids of the *Ct*PL-DM-expressing sequence to mimic those in *Is*PETase (Fig. 1b). As a result, transformation of the modified *Ct*PL-DM construct led to successful expression of the recombinant protein in *P. pastoris* (see below).

### Restoration of PET hydrolytic activity of *P. pastoris*-expressed *Ct*PL-DM

**Fig. 2:**
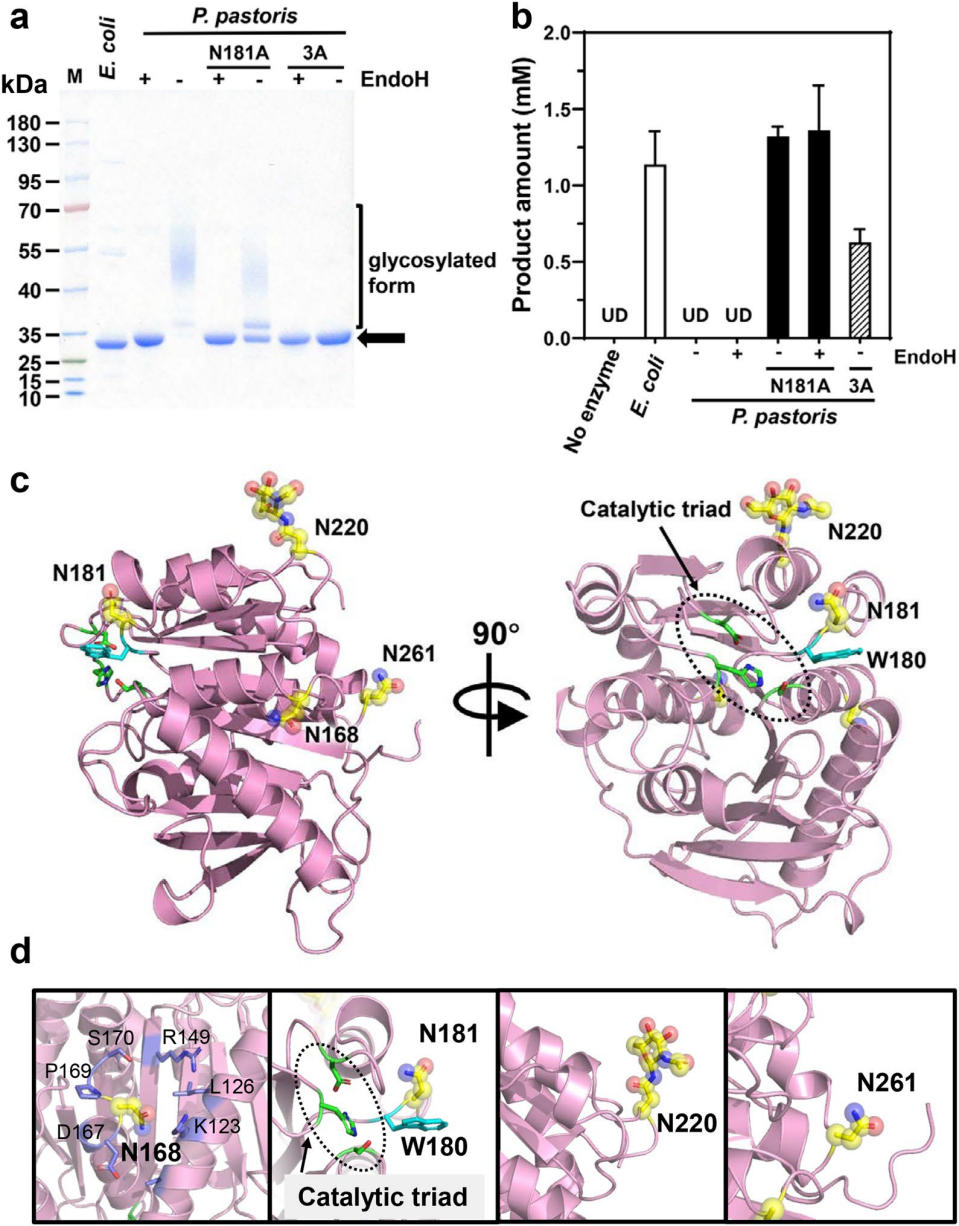
The effects of *N*-glycosylation on *P. pastoris*-expressed *Ct*PL-DM. **a** SDS-PAGE analysis of recombinant proteins expressed in *E. coli* and secreted by *P. pastoris* with or without EndoH treatment. Lane M: protein marker. The theoretical migration distance of *Ct*PL-DM on SDS-PAGE is indicated by an arrow. **b** PET hydrolytic activity of *Ct*PL-DM and each variant expressed by *E. coli* and *P. pastoris* that were treated with or without EndoH. The hydrolytic products (MHET and TPA) released from GfPET film by each enzyme as well as blank control without enzyme were measured at 18 h. A triplicate assay was conducted and the average values ± standard deviation are presented. UD, undetectable; 3A, N181A/N220A/N261A. **c** Crystal structure of *Ct*PL-DM (PDB ID, 8IAN), *N*-glycosylation sites are indicated as yellow sticks and transparency spheres with glycan colored by elements. The catalytic triad residues (black dashed circle) are shown as sticks. **d** The zoom-in views of four putative *N*-glycosylation sites

Although the recombinant protein of *Ct*PL-DM was successfully expressed in *P. pastoris*, we surprisingly found that the protein exhibited no detectable PET hydrolytic activity (Fig. 2a, b and Additional file 1: Fig. S1a). One major discrepancy between *E. coli*- and *P. pastoris*-expressed protein is the latter contains high degree *N*-glycosylations (Fig. 2a). In our previous report, de-glycosylation treatment with EndoH can significantly enhance the PET hydrolytic activity of *P. pastoris*-expressed *Is*PETase and *Bur*PL-DM (Chen et al. 2021). We then treated *P. pastoris*-expressed *Ct*PL-DM with EndoH and still did not detect PET hydrolytic products (Fig. 2b and Additional file 1: Fig. S1a). We thus proceeded to solve the crystal structure of *P. pastoris*-expressed *Ct*PL-DM to explore the possible reason underlying the loss of activity. We obtained the *apo*-form crystal structures of wild type and inactive variant S155A of *Ct*PL-DM (Table 1), which adopt the canonical α/β-hydrolase fold and are highly identical to the structure of *Is*PETase (Cα RMSD 0.477 Å and 0.471 Å) (Additional file 1: Fig. S2). The catalytic triad was found in an open cleft formed on the protein surface (Fig. 2c). The TPA-binding W180 adopts the C-type conformation in the *Ct*PL-DM structure and an alternative type in *Ct*PL-DM-S155A (Additional file 1: Fig. S2). This suggests that DM strategy has conferred the TPA-binding Trp in *Ct*PL-DM a wobble conformation.

**Table 1:** Data collection and refinement statistics of *Ct*PL-DM crystals

	*Ct*PL-DM	*Ct*PL-DM-S155A	*Ct*PL-DM-N181A-F235L-S155A
Data collection			
Space group	*P*3_2_21	*P*2_1_2_1_2	*I*222
Unit cell			
*a, b, c* [Å]	115.4/115.4/94.7	65.3/153.9/53.0	43.8/62.4/157.2
*α*/*β*/*γ* (°)	90/90/120	90/90/90	90/90/90
Resolution [Å]	37.76–2.08 (2.11–2.08)^a^	36.29–2.14 (2.27–2.14)	33.61–1.92 (1.95–1.92)
Unique reflections	44,073 (1813)	30,315 (1224)	16,729 (731)
Redundancy	8.49 (6.53)	9.32 (6.15)	6.46 (4.61)
Completeness [%]	100.0 (100.0)	99.9 (100.0)	98.7 (95.3)
Average I/σ (I)	9.86 (2.04)	12.52 (3.79)	14.69 (3.98)
*R*_merge_ [%]^b^	13.8 (59.3)	11.62 (36.77)	8.23 (26.73)
Refinement^c^			
No. of reflections	41,816 (3070)	28,774 (2074)	15,834 (1117)
*R*_work_ (95% of data)	0.200 (0.292)	0.169 (0.199)	0.197 (0.246)
*R*_free_ (5% of data)	0.254 (0.324)	0.220 (0.237)	0.262 (0.291)
r.m.s.d. bonds [Å]^d^	0.009	0.009	0.008
r.m.s.d. angles [º]	1.60	1.61	1.56
Ramachandran statistics^e^			
Most favored [%]	97.8	97.4	98.8
Allowed [%]	2.1	2.6	1.2
Disallowed [%]	0.2	0	0
No. of non-H atoms / Average B [Å^2^]			
Protein	4118/27.1	4137/18.4	1963/23.5
Water	512/34.8	458/28.6	142/27.9
Ligand	30/50.2	28/29.5	–
*PDB ID code*	8IAN	8IBI	8IBJ

^a^Values in parentheses are for the highest resolution shell

^b^*R*_merge_ = ΣhklΣi|*I*i(*hkl*)-〈*I*(*hkl*)〉|/ΣhklΣi*I*i(*hkl*), in which the sum is over all the *i* measured reflections with equivalent Miller indices *hkl*;〈I(*hkl*)〉is the averaged intensity of these *i* reflections, and the grand sum is over all measured reflections in the data set

^c^All positive reflections were used in the refinement

^d^According to Engh and Huber (Engh and Huber 1991)

^e^Calculated by using MolProbity (Chen et al. 2010)

In both structures, a sugar that is covalently linked to N220 was observed (Fig. 2c and Additional file 1: Fig. S3). Protein sequence analysis indicates that there are three putative *N*-glycosylation sites in addition to N220 including N168, N181 and N261 (Additional file 1: Fig. S4). As revealed by the crystal structures, the side chain of N168 is surrounded by several neighboring residues and might be inaccessible to the glycosylation machinery (Fig. 2d). N181 and N261 face towards the bulk solvent and could be glycosylated, despite no additional electron density map attached to these residues was observed (Fig. 2d). Notably, N181 is located adjacent to the TPA-binding residue W180 (Fig. 2d), and its glycosylation, if any, could likely impose spatial hindrance to prevent W180 from wobbling and impact the substrate binding. Although EndoH is capable of hydrolyzing glycosidic bonds to remove most of the glycosylations on protein surfaces, at least one residual sugar that is covalently attached to the Asn residue is retained. We wonder whether the glycosylated N181 would influence *Ct*PL-DM activity, thus constructed variant N181A and had its activity measured. SDS-PAGE analysis clearly indicated that N181 is glycosylated as the variant N181A showed lower degree of glycosylation than the parental enzyme (Fig. 2a). Furthermore, the *P. pastoris*-expressed N181A exhibits PET hydrolyzing activity that is comparable to that of *E. coli*-expressed protein (Fig. 2b and Additional file 1: Fig. S1b). Notably, de-glycosylation did not further elevate N181A performance, suggesting that the other *N*-glycosylation sites might play minimal roles in *Ct*PL-DM-catalyzed PET degradation. Indeed, depletion of all three putative *N*-glycosylations sites (3A, N181A/N220A/N261A) did not further benefit the enzyme reactivity (Fig. 2b).

### Engineering the N181A variant

**Fig. 3:**
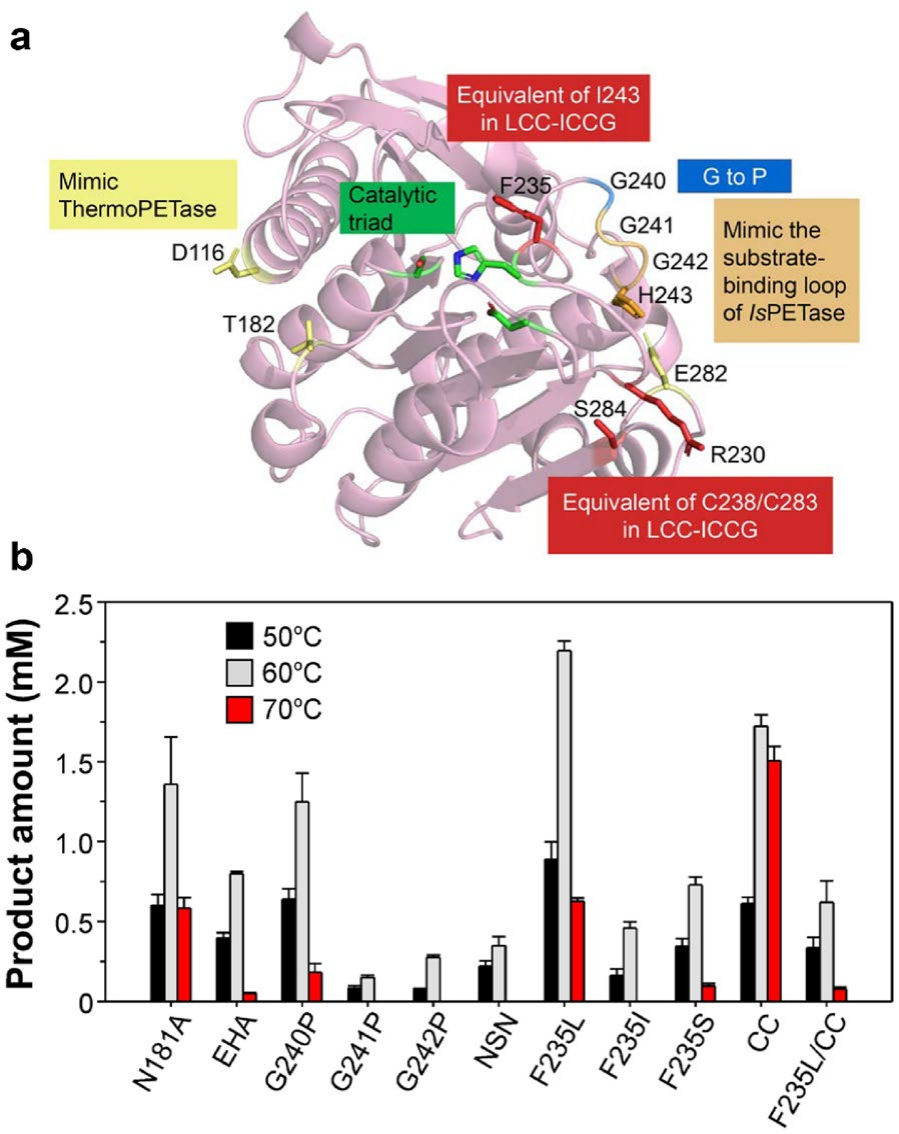
Engineering of variant N181A. **a** The overall structure of *Ct*PL-DM (PDB ID, 8IAN) in cartoon representation. Residues selected for engineering are displayed in sticks, with each color correspond to the mutation strategies indicated in the colored box. The residues comprising the catalytic triad (green box) are shown as sticks. **b** De-glycosylated *P. pastoris*-expressed recombinant protein of each variant were incubated with GfPET film at indicated temperature for 18 h. The total amount of hydrolytic products (MHET and TPA) were measured and presented as shown

We next set to enhance the performance of N181A through conducting molecular engineering (Fig. 3a). First, we compared the protein sequence of N181A and the thermostable variant of *Is*PETase (*Is*PETase^EHA^) (Son et al. 2019) and introduces three equivalent mutations to construct an EHA variant. Second, we modified a flexible loop that contain three consecutive Gly residues and is close to the active site by replacing each Gly with Pro in an attempt to enhance protein stability (G240P, G241P and G242P). This loop was also engineered by introducing triple mutation G241N/G242S/H243N (termed NSN) to mimic *Is*PETase. Next, the strategy utilized to generate a recently reported variant of a leaf compost cutinase designated LCC-ICCG that exhibits superior PET hydrolytic activity was also applied. The enhanced PET hydrolytic activity of LCC-ICCG was attributed to the F243I modification (Tournier et al. 2020), and the I243 should expand the substrate-binding tunnel to boost the enzyme activity based on our recent report (Zeng et al. 2022). N181A also has Phe in the F243 corresponding residue 235, thus the variants F235L and F235I were constructed. In addition, F235 was altered to Ser in accordance with *Is*PETase sequence. The feature that grants LCC-ICCG higher performance at elevated temperatures is an additional pair of disulfide bond, which has also been implemented in a recently reported thermostable variant of *Is*PETase designated as HotPETase (Bell et al. 2022). Therefore, a N181A variant termed CC that carries R230C/S284C double mutation to introduce an equivalent disulfide bond was also generated. PET hydrolytic activity measurement indicates that F235L and CC are the most promising variants, whose activity was elevated by more than 60% and ~ 20% activity at 60 ºC, respectively (Fig. 3b). Notably, CC displays better performance at a higher temperature, such that more than 2.5-fold higher activity in comparison with the parental enzyme was detected at 70 °C (Fig. 3b). We also combined these two variants but did not obtain better results. Therefore, the following analyses will be focused on variant F235L.

### Structural investigation of F235L

**Fig. 4:**
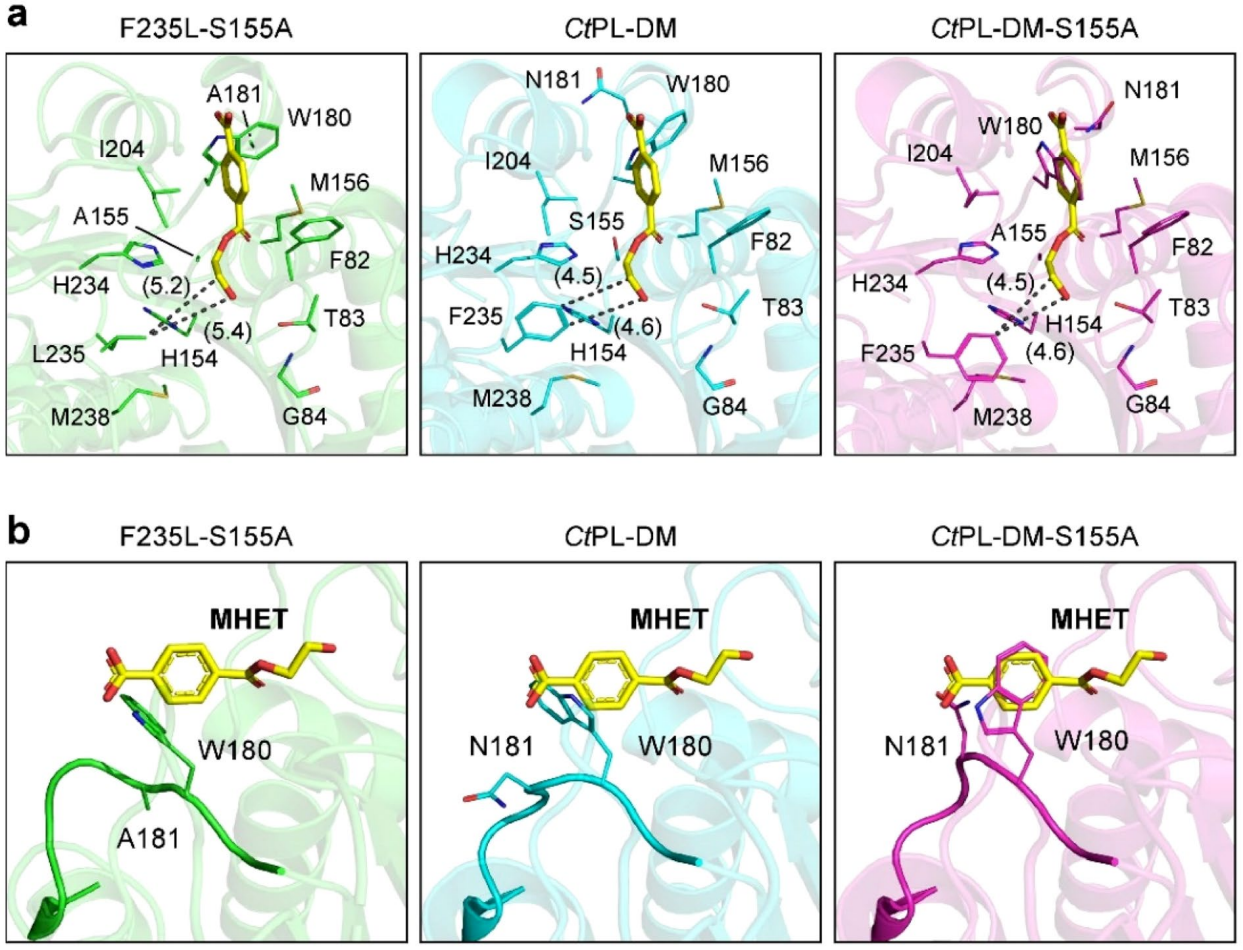
Structural comparison of *Ct*PL-DM variants. The MHET (yellow sticks) modeled from *Is*PETase/MHET complex structure (PDB ID,7XTW) were displayed in F235L-S155A (PDB ID, 8IBJ), *Ct*PL-DM (PDB ID, 8IAN) and *Ct*PL-DM-S155A (PDB ID, 8IBI). The **a** MHET-enzyme interaction networks and **b** close-up views of residue 180 and 181 in these structures are displayed. Numbers in parentheses indicate the distance measured by the dashed lines

The crystal structure of the inactive variant of F235L, termed F235L-S155A, was successfully solved, which shares a highly identical overall fold to those of *Ct*PL-DM and *Ct*PL-DM-S155A. Since the attempt to obtained substrate/analogue-bound complex failed, the MHET in *Is*PETase/MHET complex structure was modeled into these *apo*-form structures to investigate their substrate-binding modes. Compared with two DM structures, the cavity in the rear of the MHET in F235L-S155A is slightly expanded (Fig. 4a), which might create a broader substrate-binding tunnel to facilitate the binding of PET. This is similar to that was observed in the complex structure of LCC-ICCG (Zeng et al. 2022) and should be considered as a factor to render the higher activity. In addition to the substrate-binding tunnel, the structure of F235L-S155A also provides an opportunity to visualize the N181A-mediated effects. In all three structures, the TPA-binding W180 adopts conformations that should collide with the MHET (Fig. 4b). Nonetheless, W180 in F235L-S155A, which carries N181A mutation, shall be able to wobble freely. On the other hand, the conformational change of W180 in *Ct*PL-DM and *Ct*PL-DM-S155A is restrained by the glycosylated N181 (Fig. 4b), thus impedes the binding of PET.

### *Ct*PL variants-mediated PET hydrolysis in a prolong time

**Fig. 5:**
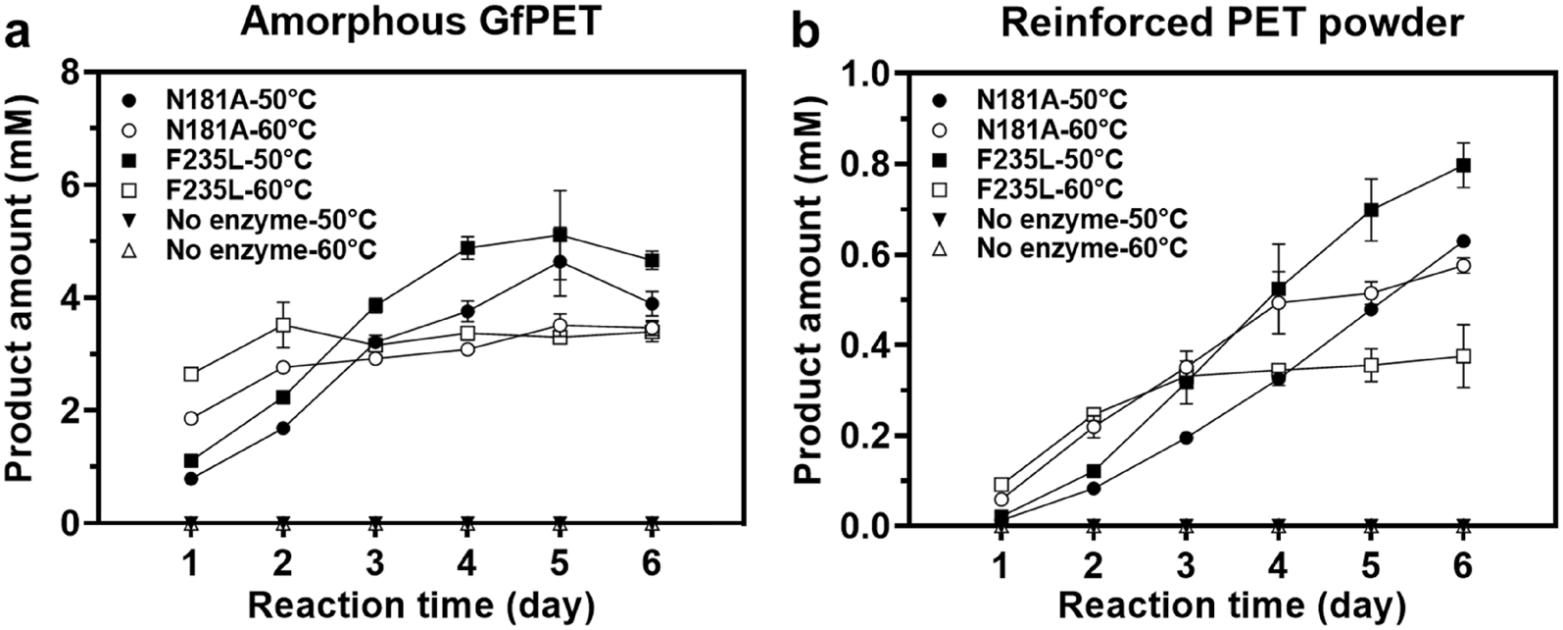
Time-dependent PET degradation activity of *Ct*PL-DM variants. Hydrolytic reactions of the two variants against **a** GfPET film and **b** reinforced PET powder, and the blank controls without adding enzyme were conducted at 50 °C and 60 °C and sampled every 24 h to measure the total amounts of hydrolytic products (MHET and TPA). The results were displayed as average values ± standard deviations

We next examined the PET hydrolytic activity of the most potent variant of *Ct*PL-DM in a prolonged reaction duration. As shown in Fig. 5a, N181A and F235L can catalyze hydrolysis of amorphous GfPET film and the hydrolytic products increased with time, with F235L higher than the parental N181A. Notably, the amounts of hydrolytic products released by N181A and F235L that operated at 60 °C reached plateaus within 3 days, while those at 50 °C reactions gradually elevated and surpassed 60 °C-treated samples in 6 days. This indicates that the optimal temperature of enzyme should be determined dependent on the reaction duration, a phenomenon similar to the previously reported results (Bell et al. 2022). The hydrolytic activities of *Ct*PL-DM variants towards a reinforced PET which contains 30% glass fiber and is more resistant to the enzyme reaction were also examined. This substrate can be decomposed by N181A and F235L though lower amounts of hydrolytic products were detected comparing to GfPET **(**Fig. 5**)**. Similarly, F235L exhibited higher activity than N181A and more hydrolytic products can be detected in 50 °C-treated samples than those of 60 °C.

## Conclusion

In this study, we demonstrated how to achieve secretory expression of *Ct*PL-DM in an industrial strain *P. pastoris*, a cutinase-like enzyme that was modified to exhibit PET hydrolytic activity. More importantly, the glycosylation modification on a residue that is located adjacent to the substrate-binding site could devastate the enzyme performance, and applying molecular engineering to remove the glycosylation site, instead of just treating the protein with de-glycosylation enzyme, is required to restore the enzyme activity. The subsequent rational design in an attempt to enhance the *Ct*PL-DM performance was conducted, which afforded a variant that harbors an expanded substrate-binding tunnel and exhibits higher activity. We also consider that the variant F235L should be a good start for further modifications and are now working on improving its thermostability via molecular engineering. Altogether, critical issues and feasible solutions in producing PET hydrolytic enzymes in *P. pastoris* are revealed in this study, and these results might be applied in other protein expression vehicles that harbor post-translational modifications. This information provides an important guidance for the development of commercial applications of biological PET recycling technologies.

### Supplementary Information

**Additional file 1: Fig. S1.** Representative HPLC chromatograms of PET hydrolytic products generated by CtPL-DM and variants. **Fig. S2.** Overall structure of CtPL-DM and the wobbling TPA-binding Trp. **Fig. S3.** N-glycosylation sites of CtPL-DM-S155A. **Fig. S4.** The full-length protein sequence of CtPL-DM. **Table S1.** Mutagenesis oligonucleotides.

## Data Availability

All data produced or analyzed and materials for this study are available in this article and its additional information files. Protein structures have been deposited in the RCSB protein data bank with ID as following: *Ct*PL-DM, 8IAN; *Ct*PL-DM-S155A, 8IBI; *Ct*PL-DM-N181A-F235L-S155A, 8IBJ.

## Abbreviations

PET: Polyethylene terephthalate
DM: Double mutation
IPTG: Isopropyl-β-D-thiogalactopyranoside
TEV: Tobacco etch virus
TPA: Terephthalic acid
MHET: Mono-(2-hydroxyethyl) terephthalate
EG: Ethylene glycol
