# Supplementary material for: Functional tailoring of a PET hydrolytic enzyme expressed in Pichia pastoris
Source: Bioresour Bioprocess. 2023 Apr 6;10(1):26. doi: 10.1186/s40643-023-00648-1 (PMC10991172; doi:10.1186/s40643-023-00648-1)
Supplement: Supplementary file 1 — Additional file 1: Fig. S1. Representative HPLC chromatograms of PET hydrolytic products generated by CtPL-DM and variants. Fig. S2. Overall structure of CtPL-DM and the wobbling TPA-binding Trp. Fig. S3. N-glycosylation sites of CtPL-DM-S155A. Fig. S4. The full-length protein sequence of CtPL-DM. Table S1. Mutagenesis oligonucleotides. [file 40643_2023_648_MOESM1_ESM.docx]

**Additional file 1**

**Functional tailoring of a PET hydrolytic enzyme expressed in *Pichia pastoris***

Xian Li^1^, Beilei Shi^1^, Jian-Wen Huang, Ziyin Zeng, Yu Yang, Lilan Zhang, Jian Min, Chun-Chi Chen*, Rey-Ting Guo*

State Key Laboratory of Biocatalysis and Enzyme Engineering, Hubei Hongshan Laboratory, Hubei Collaborative Innovation Center for Green Transformation of Bio-Resources, Hubei Key Laboratory of Industrial Biotechnology, School of Life Sciences, Hubei University, Wuhan 430062, PR China

^1^These authors contributed equally: Xian Li and Beilei Shi

*Corresponding authors. E-mail addresses: [ccckate0722@hubu.edu.cn](mailto:ccckate0722@hubu.edu.cn) (C.-C. Chen), [guoreyting@hubu.edu.cn](mailto:guoreyting@hubu.edu.cn) (R.-T. Guo)


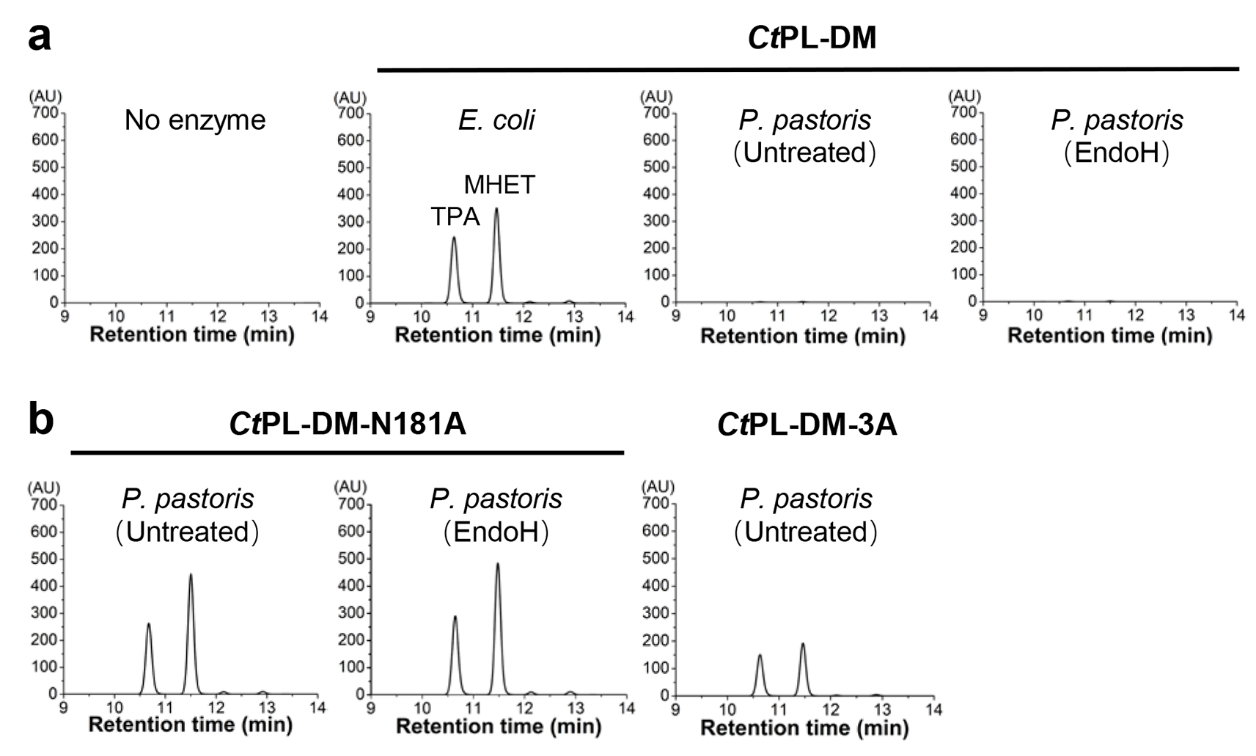


**Additional file 1: Fig. S1 Representative HPLC chromatograms of PET hydrolytic products generated by *Ct*PL-DM and variants**. (**a**) GfPET incubated with or without *Ct*PL-DM expressed in *E. coli* or *P. pastoris* under the condition described in **Fig. 2b** and analyzed by HPLC. The peaks correspond to TPA and MHET are noted. (**b**) The HPLC chromatograms of PET hydrolytic products of *Ct*PL-DM variants expressed in *P. pastoris*.


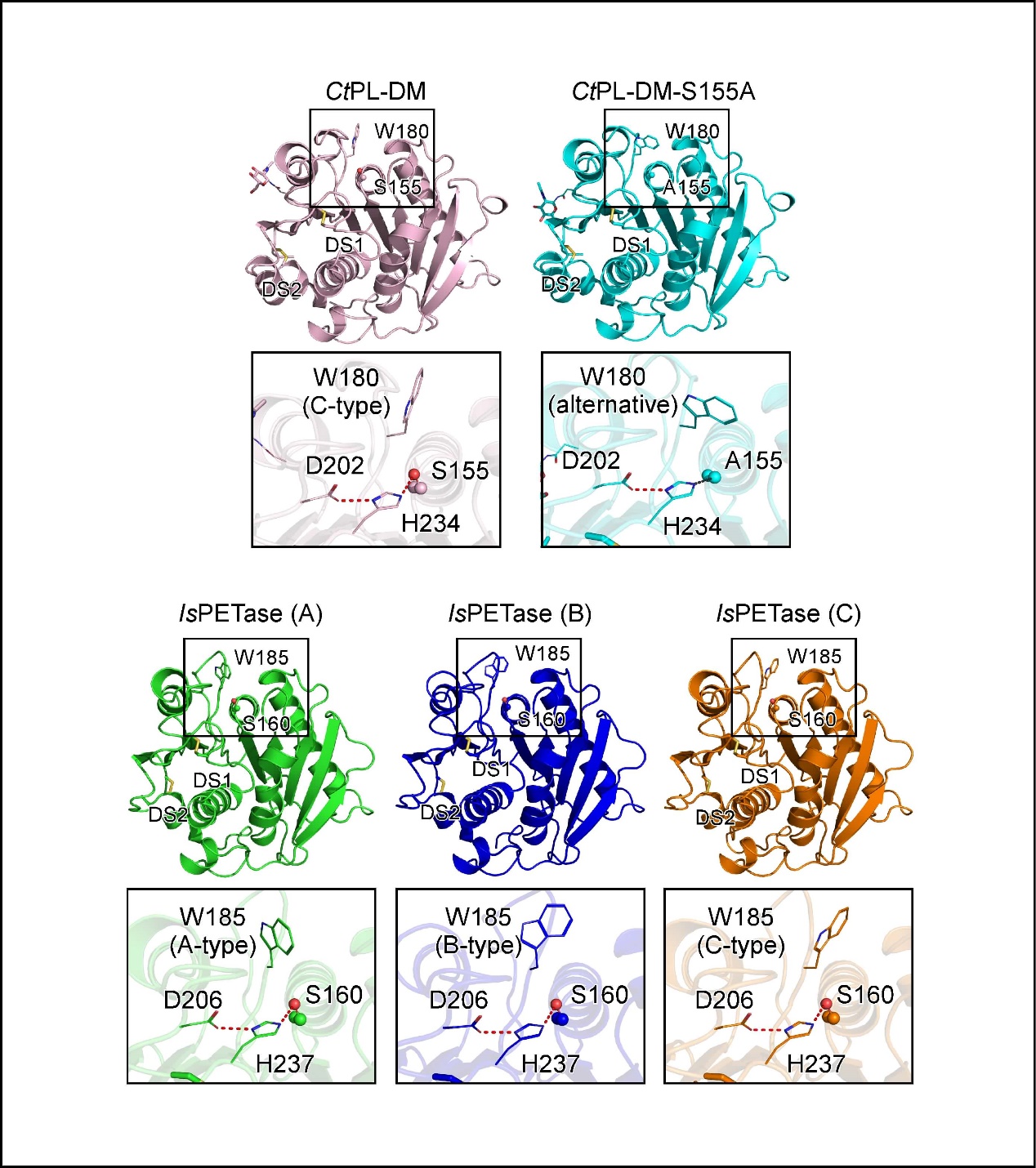


**Additional file 1: Fig. S2 Overall structure of *Ct*PL-DM and the wobbling TPA-binding Trp**. The overall structures of *Ct*PL-DM (PDB ID, 8IAN), *Ct*PL-DM-S155A (PDB ID, 8IBI) and *Is*PETase (PDB ID, 5XG0) are displayed in cartoon models. The three polypeptide chains of *Is*PETase that harbor their W185 in various conformations are individually shown and noted as A, B and C. The active centers are framed and zoomed-in below each structures. In zoom-in views, residues constituting catalytic triads and the TPA-binding Trp are shown in lines, with the catalytic residue Ser (or Ala in the inactive variant) shown in spheres. The type of Trp conformation is also denoted. DS, disulfide bond. Dashed lines, distance < 3.5 Å.


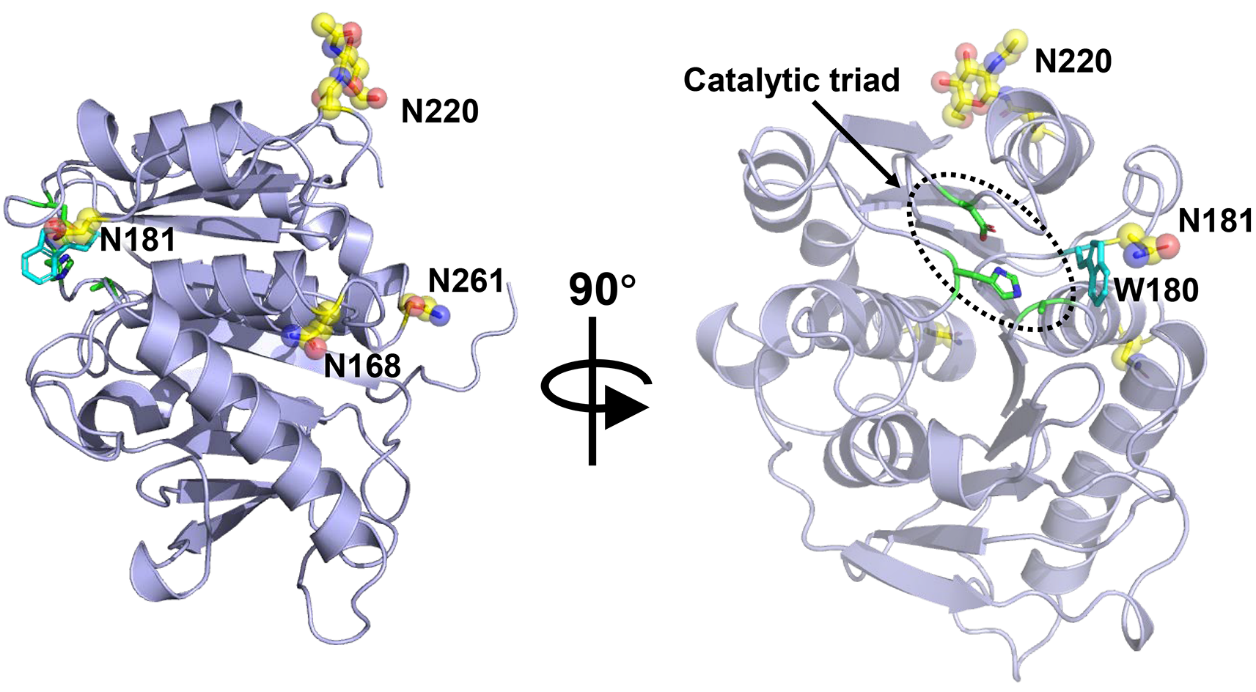


**Additional file 1: Fig. S3 *N*-glycosylation sites of *Ct*PL-DM-S155A.** Four putative glycosylation sites and a glycan that was observed in *Ct*PL-DM-S155A crystal structure (PDB ID, 8IBI) are shown as yellow sticks and transparent spheres. Two panels are related by 90 degree at Y-axis. The residues comprising the catalytic triad are shown in green sticks and circled (right panel).


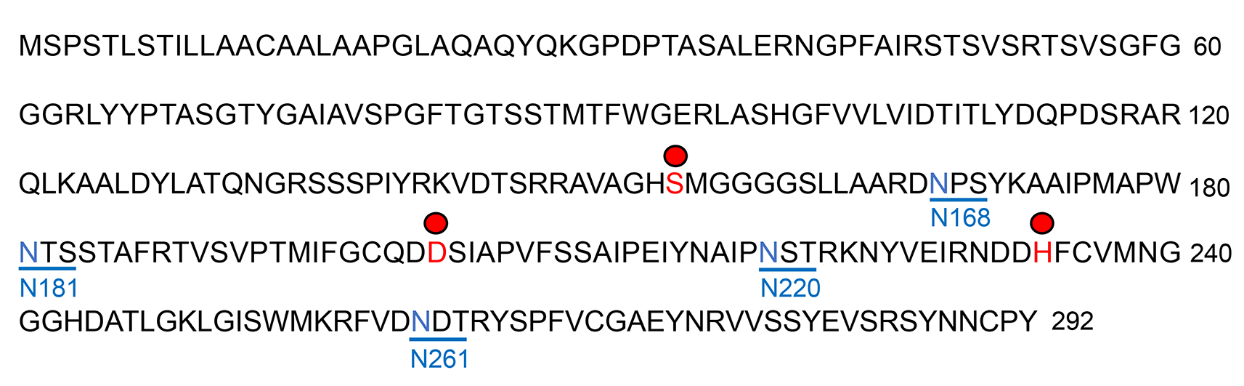


**Additional file 1: Fig. S4 The full-length protein sequence of *Ct*PL-DM.** The residues composing the catalytic triad are indicated by red circles. Four putative *N*-glycosylation sites are underlined with bold blue lines.

**Additional file 1: Table S1. Mutagenesis oligonucleotides.**

| Variant | Sequence (5’→3’) |
| --- | --- |
| *Ct*PL_DM(NP)* | GAAGCTGAATTCATGAACCCATATCAAAAAGGCCCCGATC |
| D116E | GCCCGAGAGCCGCGCCCGTCAGCTCAAGGCAGCACTGG |
| N181A | CTGGGCAACCTCGTCCACGGCCTTCCGCACGGTC |
| T182H | GAACCACTCGTCCACGGCCTTCCGCACGGTCAGCG |
| R230C | TCTGCAATGACGACCACTTCTGCGTGATGAACGGCGG |
| F235I | CACATATGCGTGATGAACGGCGGTGGGCATGATGCC |
| F235L | CACTTATGCGTGATGAACGGCGGTGGGCATGATGCC |
| F235S | CACTCTTGCGTGATGAACGGCGGTGGGCATGATGCC |
| G240P | ACCCCGGTGGGCATGATGCCACGCTGGGCAAGCTGGG |
| G241N | GCAATGGGCATGATGCCACGCTGGGCAAGCTGGGC |
| G241P | GCCCTGGGCATGATGCCACGCTGGGCAAGCTGGGCATC |
| G242P | GTCCGCATGATGCCACGCTGGGCAAGCTGGGCATCTC |
| G242S | GTTCGCATGATGCCACGCTGGGCAAGCTGGGCATCTCG |
| H243N | GGAATGATGCCACGCTGGGCAAGCTGGGCATCTCGTGG |
| E282A | GCTACGCGGTCTCGCGTTCGTACAACAACTGTCCG |
| S284C | TCTGCCGTTCGTACAACAACTGTCCGTACTGATGAGC |

* This variant contains altered N-terminal amino acids to elevate the expression level in *Pichia pastoris*.

The underlined nucleotides are mutation sites.
